# One-Health Approach to Managing Aflatoxin-Producing Aspergillus flavus Using Spent Mushroom Substrate of *Pleurotus* spp

**DOI:** 10.1021/acsomega.5c00858

**Published:** 2025-06-11

**Authors:** Wesley Morais de Araújo, Emanuel Pereira Silva, Gessymara Cainã Sales da Silva, Lívia Soares de França Silva, Francinalva Dantas de Medeiros, José Eduardo Gonçalves, Bruna Braga Dantas, Kelvyn Kennedy de Figueiredo Silva, Anauara Lima e Silva, Josean Fechine Tavares, Jhonatan Rafael Zárate-Salazar, Juliana Moura-Mendes, Fillipe de Oliveira Pereira

**Affiliations:** a Fungi Research Group, Academic Unit of Health, Education and Health Center, 154624 Federal University of Campina Grande, Cuité 58175-000, Brazil; b Planetary Health Research Group, Academic Unit of Health, Education and Health Center, 154624 Federal University of Campina Grande, Cuité 58175-000, Brazil; c Centro de Estudos e Desenvolvimento Analítico Farmacêutico da Faculdade de Farmácia 28114 da Universidade Federal de Minas Gerais, Belo Horizonte 31270-901, Brazil; d Curimataú Cancer Observatory, Academic Unit of Health, Education and Health Center, 154624 Federal University of Campina Grande, Cuité 58175000, Brazil; e Graduate Program in Natural and Synthetic Bioactive Products, Center for Health Sciences, Federal University of Paraíba, João Pessoa 58051-900, Brazil; f Graduate Program Ecology and Conservation, Federal University of Sergipe, São Cristóvão 49100-000, Brazil; g Centro Multidisciplinario de Investigaciones Tecnológicas, 187173 Universidad Nacional de Asunción, San Lorenzo 3492, Paraguay

## Abstract

Food loss represents a critical global concern. Aspergillus flavus is central to this issue, contaminating maize crops with aflatoxins. The agricultural fungicide carboxin presents significant challenges due to the rise of resistant fungal strains. This study investigates the antifungal efficacy of spent mushroom substrates from Pleurotus ostreatus (SPoS) and P. djamor (SPdS) cultivated on banana leaves, combined with carboxin (CBX), against A. flavus strains isolated from maize. The SPoS and SPdS extracts showed a low biogenic amine content and a global profile (1H NMR) of volatile oils, unsaturated fatty acids, polysaccharides, oligosaccharides, tannins, and flavonoids. A. flavus strains were not susceptible to amphotericin B and itraconazole. SGPo and SGPd exhibited fungicidal activity against all strains tested, with MIC values ranging from 1024 to 2048 mg/L. CBX demonstrated fungicidal activity with MIC values ranging from 32 to 512 mg/L. No antagonism between CBX+SPdS and CBX+SPoS was observed. SPoS and SPdS showed significant inhibition of conidial germination and mycelial growth, but CBX+SPdS and CBX+SPoS were more potent than individual agents (*p* < 0.05). SPoS and SPdS reduced conidial germination and mycelial growth by more than 70%. The study also assessed the irritation potential of these agents using the HET-CAM model, classifying them as moderate irritants. These findings support that SPdS and SPoS can potentially reduce the required dosages and frequency of application of CBX, leading to more sustainable antifungal treatments with minimized environmental and resistance risks, which aligns with a one-health approach.

## Graphical abstract

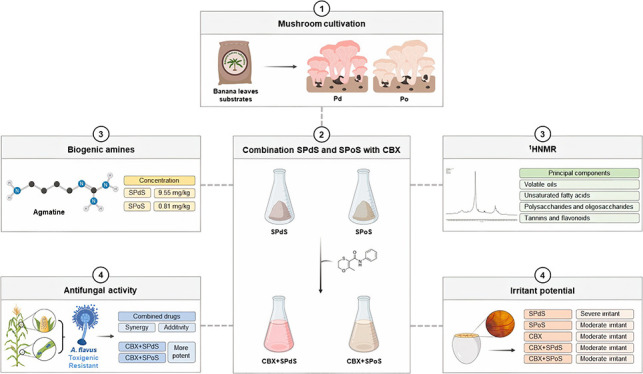

## Introduction

Fungi in agriculture are considered one of the primary biotic threats to global food loss, particularly in essential calorie and commodity crops.^1,2^ *Aspergillus* species, a group of molds with a pervasive global presence, are a significant part of this problem. *Aspergillus flavus* is an opportunistic pathogen that can infect a variety of grains, including maize, peanuts, rice, and nuts. Furthermore, it compromises food safety by producing the aflatoxins B1, B2, G1, G2, and M1, which are the most relevant from a food safety perspective due to their mutagenic and carcinogenic effects.^3^

In this context, *Zea mays* L. (maize) receives significant attention because it is highly susceptible to *A. flavus* contamination.^4^ Maize is also prominent in Latin America because it is the main staple, often featured in many traditional culinary preparations, and essential to food sovereignty and cultural identities.^5^ This underscores the urgent need for more research and policy measures to address this issue.

Fungicides are the primary tool for controlling fungal populations and reducing inoculum density, which supports their use in managing *A. flavus* in agriculture and postharvest maize grains.^6^ Carboxin (CBX) is a heterocyclic agricultural fungicide used against *Fusarium, Penicillium*, and *Aspergillus* by dysfunction of succinate dehydrogenase and the mitochondrial electron transport chain in fungal cells.^7^ Although CBX has broad-spectrum activity, it presents a medium-high risk of resistant strain emergence. *Botrytis cinerea*, *Alternaria alternata*, *Didymella bryoniae*, *Podosphaera xanthii*, and *Corynespora cassiicola* were resistant to this fungicide.^8^ The extensive use of fungicides in agriculture, including CBX, phthalimides, QoIs/strobilurins, imidazoles, and triazole, exposes environmental fungi to antifungal agents, promoting the evolution and development of resistance.^9^ As a result, consumers are at significant risk of ingesting traces of fungicide residues and drug-resistant organisms, which can render clinical treatments ineffective and pose a severe threat to public health.^10^

The increasing prevalence of antimicrobial resistance is essential to the one-health approach. Studies have shown that azole antifungals fail to inhibit environmentally induced azole-resistant isolates of *A. fumigatus, A. flavus*, and *A. terreus*. Infections caused by these azole-resistant *Aspergillus* species may be untreatable with currently available drugs, resulting in elevated morbidity and mortality rates.^11^ To achieve a one-health approach, we present for the first time the application of sustainable and natural products for controlling *A. flavus* infection: the spent mushroom substrate of *Pleurotus* ostreatus (SPoS) and *P. djamor* (SPdS) cultivated on banana leaves.

Spent mushroom substrate (SMS) is a byproduct of the cultivation of edible mushrooms with a wide range of compositions, including substrates and fungal mycelium. Large quantities of SMS byproducts can be generated, and improper disposal of SMS can lead to serious environmental problems such as soil contamination and air and water pollution, underscoring the importance of proper disposal methods.^12^ SMS biotechnological application as an antifungal agent has not yet been widely studied. We hypothesize that SMS derived from *Pleurotus* species may exhibit antifungal activity against *A. flavus*, offering an alternative for integrated fungal control.

Given this background, this study aimed to evaluate the antifungal potential of SPoS and SPdS against aflatoxigenic strains of *A. flavus* isolated from maize. Specifically, we investigated the individual and combined effects of SPoS, SPdS, and CBX on fungal growth and viability and their cytotoxicity profiles. Our findings seek to contribute to sustainable biocontrol strategies, promoting circular economy principles and aligning with one-health goals in managing fungal contamination and resistance.

**Table 1:** Characteristics of the Aspergillus flavus Strains Used in This Study^a^

						mycotoxigenic profile (MALDI-TOF MS)		GenBank accession number	
species	source	code	origin	ITS	calmodulin	AFB	AFG	ITS	CaM
Aspergillus flavus	Avatí-morotí maize grains	As 27	Paraguay	MN481412	MN604185	AFB2	AFG1	MN481412	MN604185
Aspergillus flavus	Avatí-morotí maize grains	As 29	Paraguay	MN481413	MN604186	AFB1	ND	MN481413	MN604186
Aspergillus flavus	Avatí-morotí maize grains	As 35	Paraguay	MN481414	MN604187	AFB1	ND	MN481414	MN604187
Aspergillus flavus	Avatí-morotí maize grains	As 84	Paraguay	MN423342	MN393232	ND	AFG1	MN423342	MN393232
Aspergillus flavus	Avatí-morotí maize grains	As 96	Paraguay	MN423340	MN416021	ND	AFG1	MN423340	MN416021
Aspergillus flavus	Avatí-morotí maize grains	As 101	Paraguay	MN478362	MN443169	ND	AFG2	MN478362	MN443169
Aspergillus flavus	Avatí-morotí maize grains	As 103	Paraguay	MN478363	MN443170	ND	AFG1	MN478363	MN443170
Aspergillus flavus	Avatí-morotí maize grains	As 118	Paraguay	MN478364	MN443171	AFB1	ND	MN478364	MN443171

a ND: not detected. AFB: aflatoxin B. AFG: aflatoxin G.

## Results

Initially, the SPdS and SPoS extracts were chemically characterized by their global chemical profiling (Figures 1, 2, and 3) and biogenic amines. Nonuseful regions of the ^1^H NMR spectra of SPdS and SPoS were removed to avoid interference in data analysis. No distortion of the spectral signals was observed after aligning the spectra in the new data set, demonstrating that peak shifts were satisfactorily corrected (Figure 1A). The spectra were divided into four regions: R1, R2, R3, and R4 (Figures 2 and 3). The spectra of the extracts appeared visually similar. Intense signals were observed between 0.5 and 1.7 ppm (R1), which correspond to the presence of alkyl protons from methyl (CH_3_) or methylene (CH_2_)_n_ groups not attached to aromatic rings or carboxylic groups (Figures 2 and 3, Table 02). We observed the presence of signals in the region between 1.8 and 2.9 ppm (Figures 2 and 3, R2), which are related to protons attached to carbons bonded to aromatic rings and/or functional groups, such as fatty acid carbonyls, for example (Figures 2 and 3). There was a significant overlap of signals in the region between 3.0 and 5.5 ppm (R3) and signals between 5.6 and 8.5 ppm (R4) (Figures 2 and 3). These signals are characteristic of protons from alcohols, ethers bonded to oxygenated carbons, anomeric protons, and protons belonging to glycosidic units. Finally, tannins and flavonoids show resonant signals in these more unprotected spectra regions. Only putrescine (0.40 mg/kg in SPdS), cadaverine (1.58 mg/kg in SPdS), and agmatine were detected among the amines analyzed. Agmatine was the most abundant in SGPd and SGPo, with 9.55 mg/kg values and 0.81 mg/kg, respectively.

**Figure 1:**
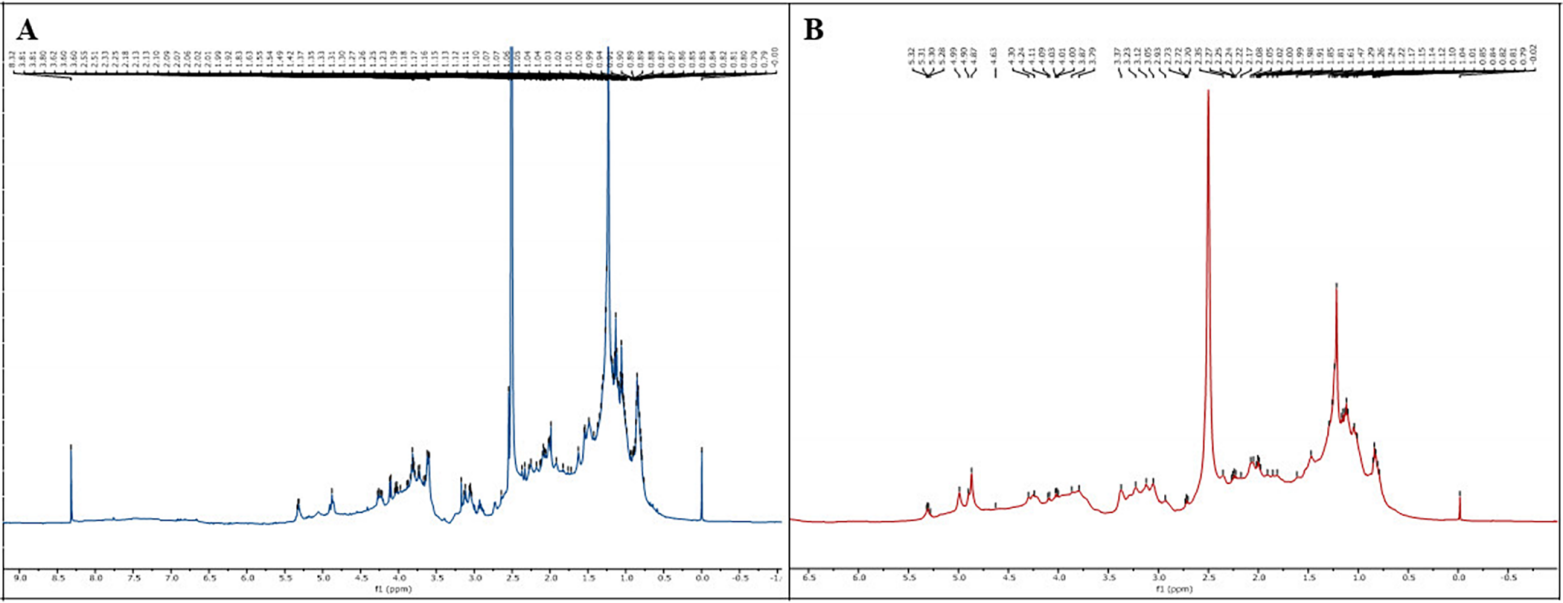
^1^H NMR spectra of (A) spent Pleurotus djamor substrate (SPdS) and (B) spent Pleurotus ostreatus substrate (SPoS) cultivated on substrates with banana leaves.

**Figure 2:**
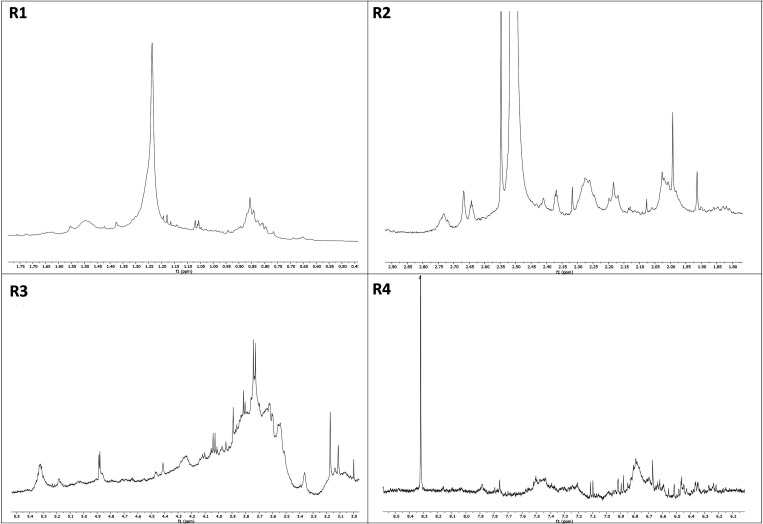
^1^H NMR spectra of spent Pleurotus djamor substrate (SPdS). R1. Region between 0.5 and 1.7 ppm. R2. Region between 1.8 and 2.9 ppm. R3. Region between 3.0 and 5.5 ppm. R4. Region between 5.6 and 8.5 ppm.

**Figure 3:**
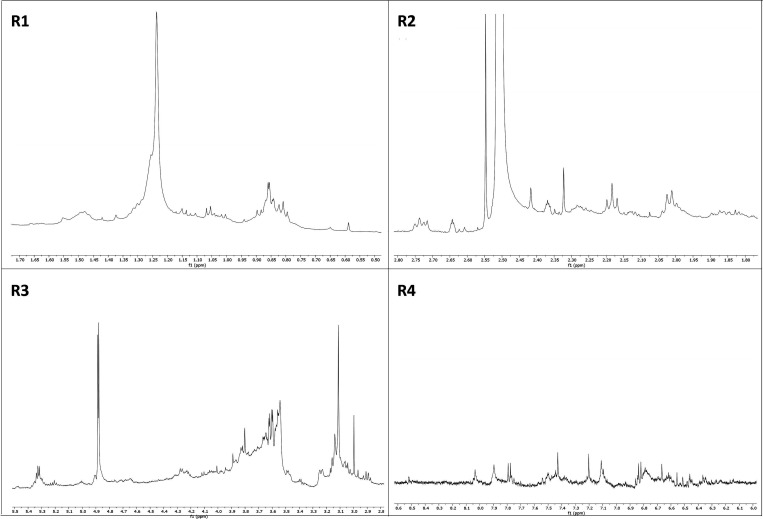
^1^H NMR spectra of spent Pleurotus ostreatus substrate (SPoS). R1. Region between 0.5 and 1.7 ppm. R2. Region between 1.8 and 2.9 ppm. R3. Region between 3.0 and 5.5 ppm. R4. Region between 5.6 and 8.5 ppm.

Table 2 shows the sensitivity profile of *A. flavus* strains. Griseofulvin and fluconazole did not inhibit the strains at the concentrations tested. Ketoconazole and miconazole inhibited 100% of the strains at 8 mg/L and 16 mg/L, respectively. The strains were most sensitive to itraconazole, with a MIC of 4 mg/L for 4 of those tested. Amphotericin B inhibited most strains at 2 mg/L. The MIC of ciclopirox was 16 mg/L for all strains. Terbinafine showed the lowest MIC values (0.5 mg/L). Table 2 shows the MIC and MFC values of SGPo and SGPd. SGPo and SGPd exhibited fungicidal activity against all strains tested, with MIC values ranging from 1024 to 2048 mg/L. CBX demonstrated fungicidal activity and had the most potent inhibitory effect against the strains, with MIC values ranging from 32 to 512 mg/L.

**Table 2:** Sensitivity Profile of Aspergillus flavus Strains to Conventional Antifungals, Spent Pleurotus djamor Substrate (SPdS), and Spent Pleurotus ostreatus Substrate (SPoS)^a^

		strains					
drugs	concentration (mg/L)	As 27	As 29	As 35	As 101	As 103	As 118
ketoconazole	MIC	8	8	4	4	4	4
	MFC	16	8	8	4	4	8
	MFC/MIC	2 (fungicide)	1 (fungicide)	2 (fungicide)	1 (fungicide)	1 (fungicide)	2 (fungicide)
miconazole	MIC	16	16	16	16	16	16
	MFC	32	16	16	16	16	16
	MFC/MIC	2 (fungicide)	1 (fungicide)	1 (fungicide)	1 (fungicide)	1 (fungicide)	1 (fungicide)
fluconazole	MIC	X	X	X	X	X	X
	MFC	X	X	X	X	X	X
	MFC/MIC	X	X	X	X	X	X
itraconazole	MIC	4	4	4	4	32	8
	MFC	4	4	4	4	32	8
	MFC/MIC	1 (fungicide)	1 (fungicide)	1 (fungicide)	1 (fungicide)	1 (fungicide)	1 (fungicide)
amphotericin B	MIC	4	2	2	2	2	2
	MFC	4	4	8	8	2	4
	MFC/MIC	1 (fungicide)	2 (fungicide)	4 (fungicide)	4 (fungicide)	1 (fungicide)	2 (fungicide)
ciclopirox	MIC	16	16	16	16	16	16
	MFC	16	16	32	128	64	32
	MFC/MIC	1 (fungicide)	1 (fungicide)	2 (fungicide)	8 (fungistatic)	4 (fungicide)	2 (fungicide)
griseofulvin	MIC	X	X	X	X	X	X
	MFC	X	X	X	X	X	X
	MFC/MIC	X	X	X	X	X	X
terbinafine	MIC	0,5	0,5	0,5	0,5	0,5	0,5
	MFC	0,5	0,5	0,5	0,5	0,5	0,5
	MFC/MIC	1 (fungicide)	1 (fungicide)	1 (fungicide)	1 (fungicide)	1 (fungicide)	1 (fungicide)
carboxin	MIC	32	32	32	512	512	512
	MFC	32	32	64	2048	2048	2048
	MFC/MIC	1 (fungicide)	1 (fungicide)	1 (fungicide)	2 (fungicide)	4 (fungicide)	4 (fungicide)
SPdS	MIC	2048	1024	1024	1024	2048	2048
	MFC	2048	4096	4096	4096	2048	4096
	MFC/MIC	1 (fungicide)	4 (fungicide)	4 (fungicide)	4 (fungicide)	1 (fungicide)	2 (fungicide)
SPoS	MIC	1024	1024	1024	1024	1024	2048
	MFC	2048	2048	1024	1024	1024	2048
	MFC/MIC	2 (fungicide)	2 (fungicide)	1 (fungicide)	1 (fungicide)	1 (fungicide)	1 (fungicide)

a X: fungal growth up to the highest concentration tested.

Considering the sensitivity profile of the strains to the extracts, we conducted a study on the combination of SGPo and SGPd with CBX. Based on the results, we calculated the fractional inhibitory concentration index (FICI) and determined the effect of combining CBX with SGPo and SGPd. The MIC values for the drugs alone, in combination, and the FICI can be seen in Table 3. The inhibitory effects of the combined drugs were higher than those of the individual drugs for 83.3% of the strains. Additivity was observed in 4 strains (66.7%), while synergism was observed in 2 strains (33.3%). No antagonism between the drugs was observed. We selected the strains As27 and As118 for subsequent stages because they showed synergism in at least one of the combinations.

**Table 3:** Association Study of Spent Pleurotus djamor Substrate (SPdS) or spent Pleurotus ostreatus Substrate (SPoS) with Carboxin (CBX) against Aspergillus flavus ^a^

	MIC alone (g/L)			MIC in combination (g/L)			MIC in combination (g/L)		
strains	SPdS	SPoS	CBX	SPdS	CBX	FICI (interaction)	SPoS	CBX	FICI (interaction)
As27	2048	1024	32	256	8	0.38 (synergism)	512	16	1.00 (additivity)
As29	1024	1024	32	128	16	0.63 (additivity)	256	16	0.75 (additivity)
As35	2048	1024	32	512	16	0.75 (additivity)	128	64	2.13 (indifference)
As101	1024	1024	512	256	128	0.50 (additivity)	1024	512	2.00 (indifference)
As103	2048	1024	512	1024	512	1.50 (indifference)	1024	512	2.00 (indifference)
As118	2048	2048	512	256	128	0.38 (synergism)	256	128	0.38 (synergism)

a MIC, minimal inhibitory concentration; FICI, fractional inhibitory concentration index.

The antifungal effect of CBX, SPoS, SPdS, CBX+SPdS, and CBX+SPoS was evaluated through conidial germination and mycelial growth assays of AS27 (Figures 4A and 5A) and AS118 (Figures 4B and 5B). All drugs, individually and in combination, significantly inhibited the germination of AS27 and AS118 conidia (*p* < 0.05). Additionally, CBX+SPdS and CBX+SPoS were more potent than the individual drugs (*p* < 0.05). On average, the single drugs inhibited the germination of AS27 conidia by 71.40%, while the combined drugs reached 86.49%. The single and combined drugs inhibited the germination of AS118 conidia by an average of 71.37% and 88.36%, respectively.

**Figure 4:**
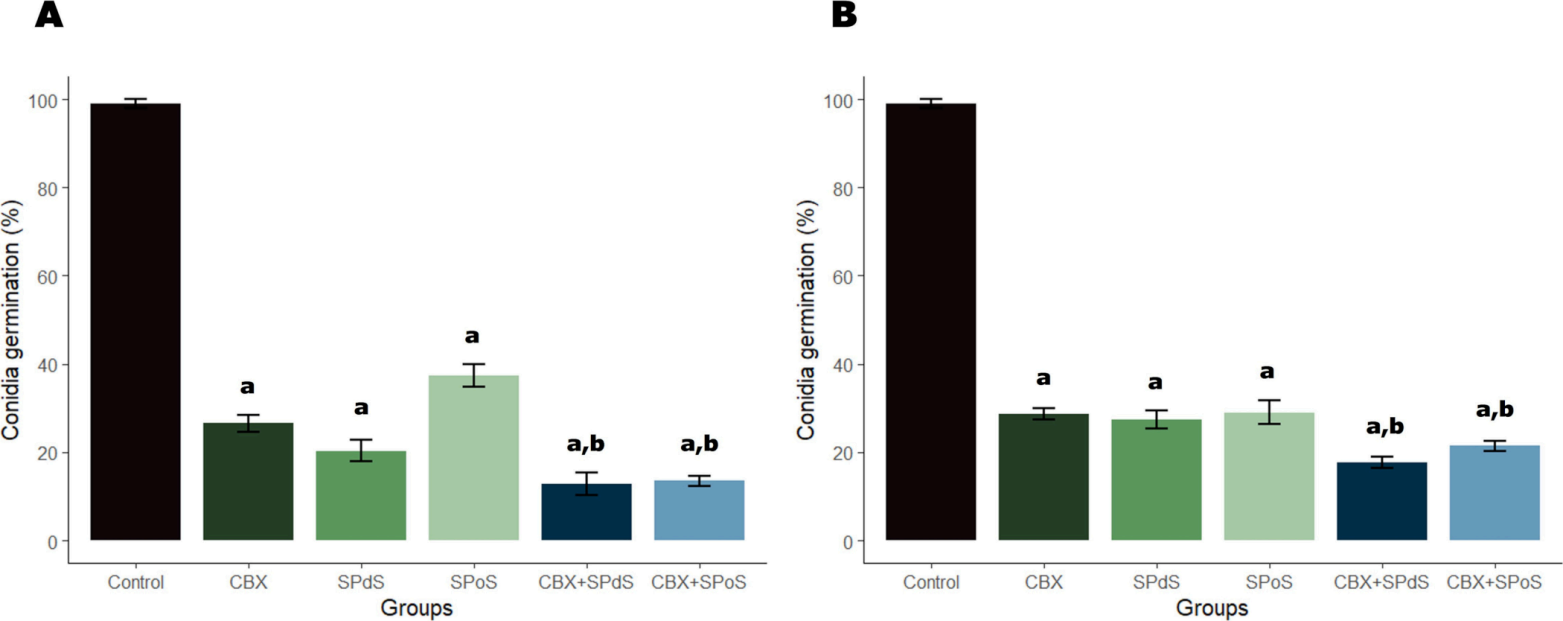
Effects of spent Pleurotus djamor substrate (SPdS), spent Pleurotus ostreatus substrate (SPoS), carboxin (CBX), and combinations on the conidia germination rate of Aspergillus flavus AS27 (A) and Aspergillus flavus AS118 (B). SPdS (MIC), SPoS (MIC), CBX (MIC), CBX+SPdS (1/2MIC + 1/2MIC), and CBX+SPoS (1/2MIC + 1/2MIC). The results are the means ± SD from three independent experiments. Significant difference (*p* < 0.05) when compared to drug-free growth control (a) and to the drugs alone (b).

**Figure 5:**
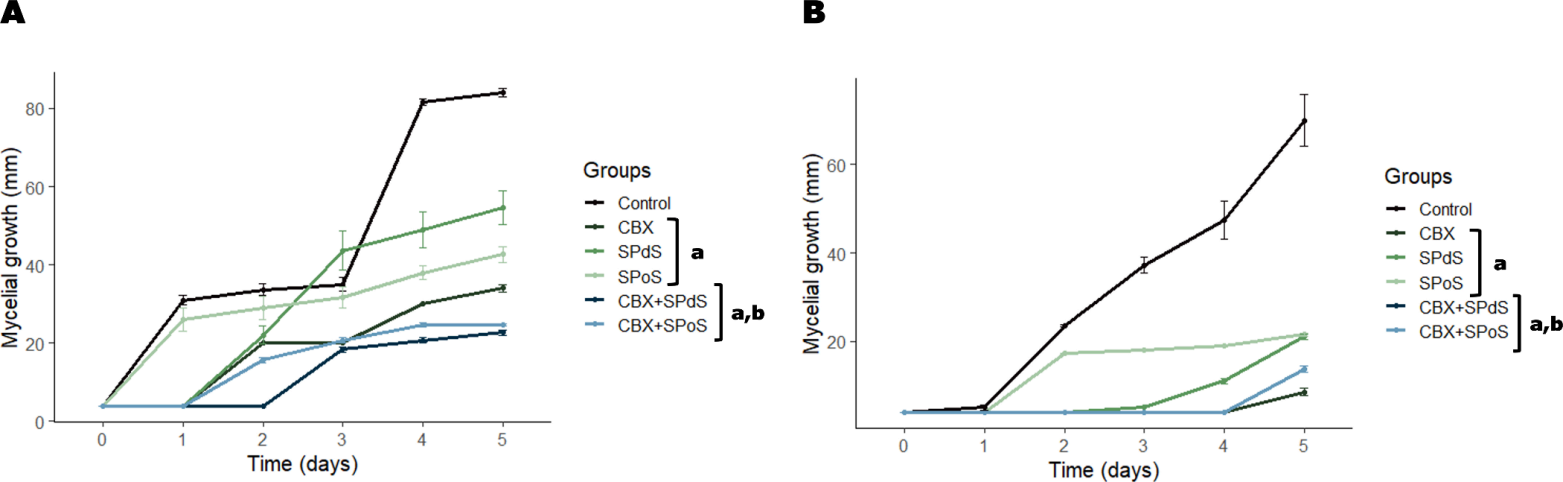
Effects of spent Pleurotus djamor substrate (SPdS), spent Pleurotus ostreatus substrate (SPoS), carboxin (CBX), and combinations on the mycelial radial growth of Aspergillus flavus AS27 (A) and Aspergillus flavus AS118 (B). SPdS (MIC), SPoS (MIC), CBX (MIC), CBX+SPdS (1/2MIC + 1/2MIC), and CBX+SPoS (1/2MIC + 1/2MIC). The results are the means ± SD from three independent experiments. Significant difference (*p* < 0.05) when compared to drug-free growth control (a) and to the drugs alone (b) on day 5.

A mixed ANOVA used in the mycelial growth assay revealed significant effects of time (*p* < 0.05) and an interaction between treatment and time (*p* < 0.05) in the mycelial growth assays (Figures 5A and B). Subsequent pairwise comparisons indicated changes in the mycelial growth of AS27 and AS118 in the control group over time. The control group showed progressive growth starting from day 2 (*p* < 0.05). Generally, fungal mycelium developed significantly after 2 days of incubation in the presence of SPdS and SPoS (*p* < 0.05). In the presence of CBX and combined drugs, this effect was only observed after 4 days of incubation. At the end of the incubation time, there were differences in the results with the drugs compared to the control (*p* < 0.05). CBX+SPdS and CBX+SPoS were more potent than the individual drugs (*p* < 0.05). On average, the single drugs reduced the mycelial growth of AS 27 by 48.56%, while the combined drugs reached 71.23%. The single and combined drugs reduced the mycelial growth of AS118 by an average of 76.20% and 85.03%, respectively.

The results concerning the irritant potential of isolated and combined SPdS, SPoS, and CBX are presented in Table 4. The data indicate that NaCl (0.9%) exhibited slight coagulation and was classified as practically nonirritant (MIS = 0.6 ± 1.1). On the other hand, applying NaOH (0.1N) to the chorioallantoic membrane caused intense vascular irritant reactions, classified as severe irritant (MIS = 18.0 ± 1.0). CBX, SPdS, CBX+SPdS, and CBX+SPoS induced hyperemia, hemorrhage, and coagulation reactions on the chorioallantoic membrane and were classified as moderate irritants. SPdS caused intense vascular events. Although SPdS (MIS = 9.8 ± 1.6) had lower MIC values than NaOH (positive control), it was classified as a severe irritant due to the intense vascular reactions observed in the assays.

**Table 4:** Irritant Potential of the Spent Pleurotus djamor Substrate (SPdS), Spent Pleurotus ostreatus Substrate (SPoS), and Carboxin (CBX) Alone and in Combination^a^

drugs	irritation score				
	hyperemia	hemorrhage	coagulation	MIS	final degree
SPdS	2.0 ± 1.2	2.7 ± 2.5	5.2 ± 2.6	9.8 ± 1.6	severe irritant
SPoS	1.6 ± 1.3	0.8 ± 1.9	4.9 ± 3.0	7.3 ± 1.9	moderate irritant
CBX	1.5 ± 1.2	2.1 ± 2.6	3.3 ± 3.5	6.9 ± 0.6	moderate irritant
CBX + SPdS	1.7 ± 1.2	1.5 ± 2.3	5.6 ± 2.0	8.7 ± 0.2	moderate irritant
CBX + SPoS	1.1 ± 1.2	0.4 ± 1.4	5.0 ± 2.5	6.5 ± 0.7	moderate irritant
NaCl 0,9%	0.1 ± 0.3	0.0 ± 0.0	0.6 ± 2.0	0.6 ± 1.1	practically nonirritating
NaOH 0,1N	5.0 ± 0.0	7.0 ± 0.0	6.3 ± 1.0	18.0 ± 1.0	severe irritant

a Results are expressed as the mean ± SD (*n* = 4). MIS (mean irritation score).

## Discussion

Although fungicides play a crucial role in agriculture and the preservation of maize grains, their use presents several disadvantages, primarily stemming from (1) overuse in food production, (2) residual toxicity from high fungicide levels in consumed foods, and (3) the development of resistant fungal strains in the environment. These factors, viewed from a one-health perspective, collectively contribute to adverse impacts on the health of animals, humans, and the shared environment. In this way, our results demonstrated that SPoS and SPdS exerted a relevant modulatory action on CBX antifungal activity *in vitro*, decreasing its effective concentration. Finally, SPdS, SPoS, and CBX were classified as moderate irritants when combined in the HET-CAM assay, indicating that the future application of these products in food protection could be feasible and relatively safe.

Recently, the EUCAST reviewed all current antifungal breakpoints for *Aspergillus* and *Candida* species.^13^ For *Aspergillus*, finding the breakpoints and the definition of the susceptibility category for the drugs amphotericin B, isavuconazole, itraconazole, posaconazole, and voriconazole is possible. We found that *A. flavus* strains were not susceptible to amphotericin B and itraconazole, which are licensed for treating systemic or severe *Aspergillus* infections. Then, the resistance profile of the *A. flavus* strains used in this study is a cause for concern once resistance in *Aspergillus* is associated with substantially increased mortality.^14^

To the best of our knowledge, the degree of exposure of the strains to carboxin and how this antifungal pressure might have driven the emergence of strains with this sensitivity profile had yet to be reported. The effects of fungicide exposure on fungal resistance to azoles have been studied in *Aspergillus fumigatus* associated with azole residues in the fields.^15^ However, studies have demonstrated the emergence of CBX-resistant strains conferred by mutations in the genes that encode subunits of the succinate dehydrogenase complex.^16^

In addition, it has established limit standards for using CBX at 0.03 mg/kg for vegetables.^17^ In Brazil, a limit of 0.05 mg/kg of CBX must be used in maize grains.^18^ Due to its application in agriculture, CBX can also accumulate in the soil, generating toxic residues that negatively impact the health and microbial diversity of the environment. Also, CBX can cause significant developmental toxicity and cardiotoxicity in zebrafish embryos, highlighting the concern for food and environmental safety.^19^ This picture underscores the need for a one-health approach to seeking lower effective doses of CBX in food production to reduce the selective pressure toward developing resistance in agricultural fields.

Combining antifungal drugs is an innovative strategy to combat antifungal resistance and reduce negative environmental impacts. To support this assessment, we used *in vitro* models of fungal development in two main growth stages: conidial germination and mycelial formation (Figures 1, 2).^20^ Our results show that SPoS and SPdS combined with CBX exhibited more promising effects than when tested individually (Figures 4, 5). This has good prospects for reduced fungicide use in agriculture and contributes to a more environmentally sustainable scenario.

The global compound profile analyzed in this study demonstrates that SPdS and SPoS contain various compounds that contribute to their antimicrobial properties, similar to data recognized in the literature for *Pleurotus* species.^21^ In this study, the signals in R1 (Figures 2 and 3) are common in mushroom extracts due to their high content of proteins, fats, carotenoids, volatile oils, phenolic compounds, tocopherols, and vitamins, which also contribute to their high nutritional value.^22^ Volatile substances found in *P. ostreatus* and *P. djamor*, such as 1-Octen-3-one and 1-Octen-3-ol, exhibit resonant signals in this region and potent antimicrobial activity against food-related bacteria and pathogenic fungi.^23^ The rich presence of unsaturated fatty acids (Figures 2 and 3, R2) in liquid extracts of mycelia and cultures of *Pleurotus* spp. has been linked to the antifungal activity of these mushrooms.^24^ The signals in R3 (Figures 2 and 3) suggest a high presence of polysaccharides and oligosaccharides, corroborating results previously reported for extracts of other mushroom species.^25^ Methanolic and aqueous extracts of *P. florida* also exhibit antifungal activity, commonly associated with flavonoids and tannins.^24^ For example, organic compounds such as p-anisaldehyde are produced by *P. ostreatus* as a defense mechanism against other organisms. Pleurotin also shows signals in more unprotected, aromatic regions and is commonly associated with antifungal and antibacterial activities.^26^

We found the antimicrobial activity of *P. ostreatus* SMS against Fusarium oxysporum f. sp. *cubense*,^27^ volatile compounds emitted by SMS of *Hypsizygus marmoreus, Pholiota microspora*, *Lyophyllum decastes*, and *Auricularia polytricha* against *Alternaria brassicicola*.^28^ However, the present study is the first evidence reporting the potential of spent mushroom substrate of *P. djamor* and *P. ostreatus* in controlling mycotoxigenic *A. flavus*.

Research indicates that SMS from *Pleurotus* species can be a safe and nutritious animal feed, particularly ruminants.^29,30^ However, SMS can be utilized as an organic fertilizer; it may pose health risks due to increased antibiotic resistance and the abundance of human disease genes.^31^ These findings suggest that while SMS has potential applications in agriculture and food production, careful consideration of safety and environmental impacts is necessary.

In this regard, we evaluated the profile of biogenic amines and the irritation potential of SPoS and SPdS to assess their future practical applications. We found negligible levels of biogenic amines in the extracts. Furthermore, SPoS and SPdS, combined with CBX, were moderate irritants in the HET-CAM model. The HET-CAM represents a suitable model to investigate the irritation potential and toxicity of substances and formulations.^32^ Some studies have used HET-CAM to evaluate the toxicity of products, focusing on foods such as a biosurfactant produced by Saccharomyces cerevisiaeURM6670 for cookies and muffins,^33^ *Dorema ammoniacum* gum resin for the food industry,^34^ and β-glucan from the mushroom *Ganoderma lucidum*.^35^

## Conclusions

This study shows a high potential of the spent substrate from *P. ostreatus* and *P. djamor* in enhancing the action of CBX as part of practices to control the growth of mycotoxigenic *A. flavus* strains. Additional field studies with other fungal agents and application models in agriculture and food are recommended. This is a notable contribution to the one-health approach, as SMS can be obtained from producing *P. ostreatus* and *P. djamor* mushrooms sustainably using agricultural residues. Furthermore, in a circular economy system, our study contributes to yet another biotechnological application of these products, which can improve food production and protection for both small and large producers.

## Materials and Methods

### Obtaining the spent mushroom substrate

The SMS of *P. djamor* (SPdS) and *P. ostreatus* (SPoS) were obtained from the cultivation of edible mushrooms of these species in substrates consisting of banana leaves (*Musa* × *Paradisiaca*) with supplements (10% wood sawdust and 10% wheat bran) as described.^36^

### Preparation of SPdS and SPoS Extracts

Samples of 2 g of fresh SPoS and SPdS were extracted by maceration with 50 mL of an ethanol/water solution (70:30 v/v) for 5 days. The extracts were then filtered using qualitative filter paper, followed by filtration with a nylon syringe filter (0.45 μm), and stored under refrigeration. The SPdS and SPoS hydroalcoholic extracts were concentrated using a vacuum rotary evaporator SL-126 to evaporate the solvent for 7 h at 50 °C, resulting in the crude hydroalcoholic extract.^37^

### Global Chemical Profiling of SPdS and SPoS Extracts

The analysis was performed by ^1^H nuclear magnetic resonance spectrometry (^1^H NMR), according to the method proposed by.^36^ Three aliquots of around 30 mg of each extract were weighed and solubilized with 550 μL of dimethyl sulfoxide (DMSO-d6, 99.96 atom % D, contains 0.03% (v/v) TMS). The solution was transferred to an NMR tube (inner diameter: 5 mm, length: 7 in.) for ^1^H NMR spectral analysis. The ^1^H NMR spectra were obtained on a Bruker ASCEND 500 MHz spectrometer (Bruker, Coventry, UK) using the following parameters: number of scans (8), gain (101.0), relaxation delay (4.6100), pulse rate (8.0000), presaturation frequency (3.36150), acquisition time (3.2768). The chemical shifts for all the samples were referenced to the DMSO-d6 solvent signal, 2.50 ppm. The spectra were processed using MestReNova (MNova) software version 14.2.0. The ^1^H NMR spectra were preprocessed using the following procedures: referencing by the solvent signal, baseline correction, phase correction, alignment, normalization by the total area of the spectrum, and removal of the solvent signal.

### Analysis of Biogenic Amines

Initially, the extraction of amines from fresh SPdS and SPoS was performed using 5% trichloroacetic acid.^38,39^ The separation of amines was conducted via ion-pair chromatography in a high-performance liquid chromatograph (HPLC) and postcolumn quantification with o-phthalaldehyde, followed by fluorescence detection at 340 nm excitation and 450 nm emission. We analyzed the presence of ten free biogenic amines: spermine, spermidine, agmatine, putrescine, cadaverine, tyramine, tryptamine, phenylethylamine, histamine, and serotonin. All standard drugs were obtained from Sigma-Aldrich (Brazil). We prepared a standard curve using a stock solution (100 μg/mL) that contained amines in hydrochloric acid (0.1 M). Working standard solutions were prepared at eight concentrations ranging from 0.5 to 30 μg/mL. For each amine, we constructed a graph of analyte signal versus concentration and calculated linear equations and correlation coefficients using ordinary least-squares regression. We spiked three samples with known amounts of amines (5, 15, and 30 μg/g) to assess precision and conducted six repetitions for each. The concentrations of the amines were determined, and recoveries were calculated.

### Drugs

In this study, we used the following antifungal drugs: carboxin, amphotericin B, ciclopirox, griseofulvin, terbinafine, fluconazole, ketoconazole, itraconazole, and miconazole. The conventional antifungals and the extracts of SPdS and SPoS were diluted at the time of testing, first dissolving them in 100 μL of dimethyl sulfoxide (DMSO) and sterile distilled water to obtain an initial concentration of 4096 mg/L (SPdS and SPoS) and 512 mg/L (conventional antifungals). Then, serial dilutions were made in RPMI 1640 medium from this concentration to achieve lower concentrations. All antifungals were obtained from Sigma-Aldrich (Brazil).

### Fungi Strains

For the antifungal activity assays, we used the following fungal strains: *A. flavus* AS27, *A. flavus* AS29, *A. flavus* AS35, *A. flavus* AS101, *A. flavus* AS103, and *A. flavus* AS118. These strains were obtained from the Microorganism Culture Collection of the National University of Asunción (CCM-UNA, Paraguay) (Table 1).^40^ The fungal inocula were adjusted with sterile distilled water to (2–5) × 10^6^ conidia/mL using a spectrophotometer at 625 nm with transmittance ranging from 70 to 80% and absorbance from 0.08 to 0.13.^41^

### Minimum Inhibitory Concentration (MIC)

MIC of the test drugs was determined using the microdilution technique.^41^ The inocula were diluted 1:10 in RPMI 1640 to obtain a working concentration of (2–5) × 10^5^ CFU/mL. A negative control (RPMI 1640 + inoculum) and a DMSO control (DMSO + inoculum + RPMI 1640) were performed. The plates were sealed and incubated at 36 °C for 48 h for reading. MIC was defined as the lowest concentration of the drugs capable of completely inhibiting fungal growth compared to the negative control.

### Minimum Fungicidal Concentration (MFC)

To obtain the MFC, volumes of 10 μL were transferred from each well without fungal growth to Sabouraud glucose agar plates. Fungal colonies were counted after incubating the plates at 28 °C for 48 h. The MFC was defined as the lowest drug concentration that resulted in no growth or fewer than three colonies (99.9% death).^42^ A drug with fungicidal action will not have an MFC/MIC ratio reaching 4, while a fungistatic drug will have an MFC/MIC ratio greater than 4.^43^

### Drug Combination Study

This step was performed by checkerboard method.^44^ We applied the following dilutions of the test drugs (8xMIC, 4xMIC, 2xMIC, MIC, 1/2MIC, 1/4MIC, and 1/8MIC) in RPMI 1640. The plates were incubated at 36 °C for 48 h to determine the MIC. The fractional inhibitory concentration index (FICI) was calculated using the following sum: FIC_A_ + FIC_B_, where A represents SPdS or SPoS and B represents CBX. FIC_A_ = Combined MIC_A_/MIC_A_ alone, while FIC_B_ = Combined MIC_B_/MIC_B_ alone. The FICI was interpreted as follows: synergy (<0.5), additivity (0.5–1.0), indifference (>1.0 and <4.0), or antagonism (>4.0). The lowest MICs of the combined drugs were used for subsequent assays.

### Conidial Germination

For conidial germination tests, sterile eppendorf tubes containing 0.5 mL of RPMI 1640 were mixed with the drugs as follows: SPdS (MIC), SPoS (MIC), CBX (MIC), SPdS (1/2MIC) + CBX (1/2MIC), and SPoS (1/2MIC) + CBX (1/2MIC). Subsequently, 100 μL of the inoculum (AS27 and AS118) was homogenized to the tubes. The tubes were then incubated at 36 °C for 48 h. A control experiment without the addition of drugs was conducted. The quantities of germinated and nongerminated conidia were determined using a hemocytometer. The germination rate was calculated for each tested group.^45^

### Mycelial Growth

We analyzed the growth using the radial mycelial growth technique on a solid medium.^46^ On Petri dishes containing 10 mL of Sabouraud glucose agar, the drugs were added as follows: SPdS (MIC), SPoS (MIC), CBX (MIC), SPdS (1/2MIC) + CBX (1/2MIC), and SPoS (1/2MIC) + CBX (1/2MIC). After solidification of the medium, freshly cultivated colonies (AS27 and AS118) on Sabouraud glucose agar (4 mm in diameter) were placed on the surface of the plates. A control experiment without the addition of drugs was conducted. The plates were then incubated at 36 °C for up to 5 days, and the radial growth diameter of the fungi was recorded daily. The results were expressed as colony diameter (mm) over the incubation time.

### Irritant Potential Assessment

The irritant potential assessment was conducted according to the Hen’s egg test on the chorioallantoic membrane (HET-CAM) method, as described by (Luepke, 1985), with minor modifications. Pathogen-free, fertilized eggs from Gallus gallus *domesticus* were acquired, individually sanitized with 70% ethanol, and maintained horizontally in an incubator with an automatic rotation system (Chocadeiras Golden, Brazil) at 37.5 ± 0.5 °C and 50 ± 1% relative humidity. After 9 days of incubation, the rotation was halted, and the eggs were removed from the incubator and inspected using ovoscopy to select fertilized eggs. Viable eggs were then reinserted into the incubator vertically, with the air chamber facing upward. On the 10th day of incubation, a circular opening was made in the eggshell at the air chamber region; the eggshell membrane was moistened with 1 mL of NaCl (0.9%) and carefully removed to expose the chorioallantoic membrane fully.

Aliquots of 300 μL of the drugs were applied to the surface of the chorioallantoic membrane as follows: SPdS (MIC), SPoS (MIC), CBX (MIC), CBX+SPdS (1/2MIC + 1/2MIC), CBX+SPoS (1/2MIC + 1/2MIC). After 20 s of exposure, the chorioallantoic membrane was rinsed with 5 mL of NaCl (0.9%), and the occurrence of vascular phenomena such as hemorrhage (He), hyperemia (Hy), and coagulation (opacity and/or thrombosis) (Co) was monitored for 5 min. NaOH (0.1N) was used as a positive control, and NaCl (0.9%) as a negative control. Each detected vascular event was graded based on its time of appearance (hyperemia, hemorrhage, coagulation (opacity and/or thrombosis).^48^ The following equation calculates the mean irritation score (MIS). The subscripts indicate the evaluated replicate. Hi denotes hyperemia, He denotes hemorrhage, and Co denotes coagulation.

$$\mathrm{MIS}=[({\mathrm{He}}_{1}+{\mathrm{Hy}}_{1}+{\mathrm{Co}}_{1})+({\mathrm{He}}_{2}+{\mathrm{Hy}}_{2}+{\mathrm{Co}}_{2})+({\mathrm{He}}_{3}+{\mathrm{Hy}}_{3}+{\mathrm{Co}}_{3})+({\mathrm{He}}_{4}+{\mathrm{Hy}}_{4}+{\mathrm{Co}}_{4})]/4$$

The final degree of the drugs was determined based on the arithmetic mean of the irritation scores obtained.^47^ All embryos were euthanized following the AVMA Guidelines for the Euthanasia of Animals.^49^ This was achieved by freezing all the eggs at −20 °C, followed by appropriate disposal.

### Statistical Analysis

The experiments were performed in three independent experiments. MIC and MFC values were expressed as mode, and conidia germination rate and mycelial growth results were expressed as mean ± SD (standard deviation). Initially, we applied the Shapiro–Wilk test for normality and the Lavene test for homogeneity of variance as presupposes. We performed one-way ANOVA with post hoc Tukey’s test for conidia germination assay to determine significant differences between the treatments. We performed mixed ANOVA with Bonferroni test correction for the mycelial growth assay. We considered that there was a statistical difference when *p* < 0.05. The confidence interval was 95%. Data treatment was performed in R 4.1.0 in the RStudio interface, and we used the ggplot2 package for data visualization.
